# Medication adherence among cardiac patients in Khartoum State, Sudan: a cross-sectional study

**DOI:** 10.5830/CVJA-2017-016

**Published:** 2017

**Authors:** Abdelmoneim Awad, Nahid Osman, Siham Altayib

**Affiliations:** Department of Pharmacy Practice, Faculty of Pharmacy, Kuwait University, Kuwait; Department of Pharmacy Practice, Faculty of Pharmacy, Qassim University, Saudi Arabia; Department of Clinical Pharmacy, Faculty of Pharmacy, National University, Khartoum, Sudan

**Keywords:** non-adherence, adherence, cardiac patients, cardiovascular medications, Khartoum State, Sudan

## Abstract

**Introduction:**

Non-adherence to medication among cardiac patients is often the major risk factor for poor clinical outcomes, increased mortality rates and higher healthcare costs. The literature evaluating the prevalence of and reasons for non-adherence in resource-poor settings is extremely limited compared to resource-rich settings. There is a scarcity of data about medication adherence in Sudan hence this study was performed to identify prevalence, predictors and barriers of non-adherence to medication among cardiac patients in Khartoum State.

**Methods:**

A descriptive, cross-sectional survey was performed using a pre-tested, self-administered questionnaire on a sample of 433 randomly selected cardiac patients attending the largest three cardiac centres located in Khartoum State. Descriptive and multivariate logistic regression analyses were used for data analysis.

**Results:**

The response rate was 89.1%. The mean (± SD) number of chronic diseases among respondents was 2.3 (± 1.3) and that of medication use was 4.2 (± 1.9). The mean (± SD) duration of medication use among participants was 6.4 (± 5.4) years. Optimal adherence was defined as having a score of greater than six on the eight-item Morisky medication adherence scale. Using this cut-off point, 49% (95% CI: 43.9–54.1) of respondents had optimal adherence and 51% (95% CI: 45.9–56.1) had poor adherence. Respondents with a high level of education, low and middle income levels, and those taking five or more medications daily were found to be significantly more non-adherent to medication use than those with low to intermediate education levels (p < 0.001), those with high income levels (p < 0.001), and those taking one to four medications daily (p = 0.039). The top four barriers for poor medication adherence among the study participants were the high cost of drugs, polypharmacy and lack of pharmacist and physician communication with patients about their drug therapy.

**Conclusions:**

The current findings highlight the need for urgent, multifaceted interventions, given the burden of cardiovascular diseases and the clinical and economic consequences of medication non-adherence. These interventions include affordable medications, easy-to-use medication regimens with fewer daily doses, ongoing communication between patients and healthcare providers, and improvement of the patient– provider partnership.

## Introduction

Cardiovascular diseases (CVDs) are the leading cause of deaths in both developed and developing countries. There is a rapid increase in the burden of CVDs in Africa and it is currently a public health concern. In Sudan, CVDs are estimated to cause 12.0% of all mortalities.^1^ Cardiovascular diseases have been consistently reported as one of the top 10 causes of hospital mortality in Sudan.^2^

Cardiovascular disease is one of the most preventable causes of death in the world, due to the fact that the majority of its risk factors are preventable or controllable.^3^ In 2006, a survey of risk factors for coronary heart disease among the population in Khartoum State showed a high prevalence of low physical activity (86.6%), overweight and obesity (53.9%), hypertension (23.6%), dyslipidaemia (19.8%), diabetes (19.2%) and smoking (12%).^2^

Non-adherence to cardiovascular medications has become increasingly documented across patient populations and cardiovascular drug classes. A meta-analysis of 20 studies involving 376 162 patients illustrates a non-adherence prevalence of 43% throughout multiple drug classes, as measured by pharmacy refill data.^4^ This is comparable to the average prevalence of non-adherence to both cardiovascular and non-cardiovascular medications in developed countries, 50% as indicated by the World Health Organisation.^5^ Non-adherence to cardiovascular medications has been associated with poor clinical outcomes, including re-admissions to hospital, subsequent myocardial infarction, increased mortality rates and increased healthcare costs. 6-8

Several studies have been conducted in developed countries to determine adherence to cardiovascular medications, and have shown the prevalence of non-adherence to cardiovascular medications, the association between non-adherence and outcomes, the reasons for non-adherence, and the interventions to improve medication adherence.^4^,^9^-^13^ By contrast, data related to the prevalence of non-adherence to cardiovascular medications from developing countries is limited, even though its prevalence in these regions is increasing at more than twice the rate observed in developed countries.^5^ A systematic review of studies conducted in developing countries shows that adherence to cardiovascular medications is suboptimal and appears comparable to that observed in developed countries. The overall adherence to cardiovascular medications pooled across studies was 57.5%.^14^

The exact prevalence rate of medication non-adherence among cardiac patients in Sudan is not known since there are limited published studies. A study was conducted at Elshaab Hospital in Khartoum to determine the adherence to secondary-prevention medication among 210 patients and it was found to be 66%.^15^ Another study was performed among 76 patients with heart failure admitted to the Sudan Heart Institute in Khartoum, which indicated that 75% of the respondents were adherent to their medications.^16^

These previous studies have used limited study populations or a small number of patients in single clinical settings and may not enable meaningful conclusions to be drawn regarding levels of adherence to cardiovascular medications. This highlights the need to expand this area of research to include patients attending multicentre, out-patient cardiovascular clinics and to improve the quality of such research. Therefore, this study was conducted to evaluate prevalence, predictors and barriers of non-adherence to medications among cardiac patients attending the three largest national referral cardiac centres located in Khartoum State.

## Methods

This was a descriptive, quantitative and cross-sectional study designed to describe the adherence of patients with cardiovascular diseases to their medications.

Sudan is one of largest countries in Africa (total area of 1 861 484 km^2^) with an estimated population of 36 million people as of July 2015 (CIA fact book, 2016). It is a federal nation consisting of 18 states. Khartoum State, the capital of Sudan, covers an area of 28 165 km2 and contains almost 20% of the population, 84% of whom live in urban areas.

This study was conducted between September 2014 and March 2015 in Khartoum State, Sudan. The study population consisted of out-patients attending the cardiac clinics in Ahmed Gasim Cardiac Surgery and Renal Transplantation Centre, Elshaab Teaching Hospital and Sudan Heart Institute, because they represent the three largest national referral cardiac centres located in Khartoum State.

The study was conducted in accordance with the Declaration of Helsinki and national and institutional standards. Ethical approval for this study was obtained from the Directorate of Research, Ministry of Health, Khartoum State. Inclusion criteria for a patient to enter the study were patients aged 18 years or older diagnosed with cardiovascular disease or its major risk factor, hypertension, who started using a cardiovascular medication for a duration of three months or more. Patients who had psychiatric disorders or cognitive impairment were excluded.

The sample size was determined using PS power and sample size calculator V.3.05.17 A sample of 260 patients would be necessary to determine a 20% difference in proportion between two groups; for example, male versus female with 90% power and at 5% significance level. Assuming a response rate of 60%, a sample size of 433 patients was approached to be included in the study. The total number of patients selected from each hospital was proportional to the out-patient population attending the hospital per year. The patients at each hospital were randomly selected, using systematic random sampling from the patients’ registration lists.

The content validity of the study questionnaire was established by a research group at Kuwait University. The questionnaire was translated into Arabic and subjected to a process of forward and backward translation. The accuracy and meaning of the translated versions both forward and backward were checked, and recommended amendments where necessary were discussed before being finalised. It was pre-tested for content, design, readability and comprehension on 16 patients with cardiovascular diseases, and modifications were made as necessary so that the questionnaire was simple to understand and answer, yet gave accurate data.

The final version of the pre-tested questionnaire was composed of four sections, and it contained both open-ended and closed questions. The first section included items to provide information about the sociodemographic characteristics of the respondents (age, gender, marital status, educational level, residence and monthly income). Section two consisted of questions to provide information about the clinical variables of the study population (type and duration of cardiovascular disease, and type and duration of medications used by the patient).

The third section evaluated adherence to medications using the validated eight-item Morisky medication adherence scale (MMAS-8).^18^ Each item measures a specific medication-taking behaviour; response categories are yes/no for each item with a dichotomous response and a five-point Likert response for the last item (never/rarely, once in a while, sometimes, usually and all the time). The negative response for each item was coded as one, except for the item asking if the patient took the medications yesterday (where a positive response was coded as one). The total score was calculated by summing the values from all eight question items. Optimal adherence was defined as having a MMAS-8 score of greater than six out of a total of eight, according to the methodology used in previous literature.19,20 Section four included questions to explore the reasons for not taking the medications regularly.

Data were collected via structured face-to-face interviews of the respondents in the waiting rooms of the cardiac clinics using the pre-tested questionnaire. The interview lasted approximately 15–20 minutes. The selected patients were contacted and given an explanation about the purposes of the research. They were assured of confidentiality and gave verbal consent to participate in the study. Data about clinical variables were checked with the attending physicians from the patients’ medical records.

## Statistical analysis

Data were entered into the Statistical Package for Social Sciences [IBM SPSS Statistics for Windows, version 23 (IBM Corp, Armonk, NY, USA)] and descriptive analysis was conducted. The results were reported as percentage (95% confidence interval) and mean (standard deviation). Univariate logistic regression was performed to determine the relationship of each independent variable with adherence to cardiac medications. All variables with p ≤ 0.25 in the univariate analysis were included in the multiple logistic regression analysis to determine the factors that were independently associated with non-adherence to cardiac medications. The excluded variables were gender (p = 0.38), marital status (p = 0.83), residence (p = 0.36), hospitals (p = 0.57) and duration of medication use (p = 0.45). Only the results of multivariate logistic analysis are reported showing odds ratio (OR) and 95% confidence interval (CI). Statistical significance was accepted at p < 0.05.

For each model, response options for the dependent variable were categorised as either ‘poor adherence’ or ‘optimal adherence’. The predictor variables were categorised as follows: (1) gender: males and females; (2) age: 18–39 years, 40–49 years, 50–59 years, ≥ 60 years; (3) marital status: married and single (includes divorced and widowed); (4) level of education: low–intermediate (0–12 years) for those who completed secondary school or less, and high (> 12 years) for those who had a diploma, bachelor degree or postgraduate degree; (5) residence: Khartoum, Khartoum North, Omdurman and outside Khartoum State; (6) hospitals: Ahmed Gasim Hospital, Elshaab Teaching Hospital and Sudan Heart Institute; (7) monthly income: low < 1 000 Sudanese pounds (SP), middle 1 000–2 000 SP, and high > 2 000 SP; (8) number of chronic diseases: one to two chronic diseases, and ≥ three chronic diseases; (9) number of medications taken: one to four medications, and ≥ five medications); (10) duration of medication use: > three months to one year, > one to five years, > five to 10 years, and > 10 years.

## Results

Table 1 summarises the sociodemographic characteristics of respondents. A total of 433 Sudanese subjects were approached to be included in the study; 386 agreed to participate, giving a response rate of 89.1%. Of the respondents, 43% were 60 years or over, 57% were females and 81.6% had low–intermediate education.

**Table 1:** Sociodemographic characteristics of the respondents (n = 386)

*Characteristic*	*Frequency (%)*
Gender	
Male	166 (43)
Female	220 (57)
Marital status	
Single*	239 (61.9)
Married	147 (38.1)
Age (years)	
18–39	79 (20.5)
40–59	42 (10
50–59	99 (25.6)
≥ 60	166 (43.0)
Educational level	
Low–intermediate education	315 (81.6)
High education	71 (18.4)
Residence (cities in Khartoum State)	
Khartoum	75 (19.4)
Khartoum North	102 (26.4)
Omdurman	92 (23.8)
Outside Khartoum State	117 (30.3)
Hospitals	
Ahmed Gasim Cardiac Surgery and Renal Transplantation Centre	110 (28.5)
Elshaab Teaching Hospital	146 (37.8)
Sudan Heart Institute	130 (33.7)
Monthly income	
Low income	140 (36.3)
Middle income	136 (35.2)
High income	110 (28.5)

*Includes divorced and widowed

Table 2 shows the clinical characteristics of the study participants. One-half of respondents had hypertension, 30.3% dyslipidaemia and 28.5% had ischaemic heart disease. The mean (± SD) number of chronic diseases among the study population was 2.3 (± 1.3) and that of medication use was 4.2 (± 1.9). Two hundred and thirty-six patients (61.1%) were using beta-blockers, and above two-fifths were using loop diuretics (47.2%), statins (47.4%), low-dose aspirin (42.7%) and warfarin (40.7%). The mean (± SD) duration of medication use among participants was 6.4 (± 5.4) years.

**Table 2:** Clinical characteristics of the respondents (n = 386)

*Characteristic*	*Frequency (%; 95% CI)*
Types of chronic diseases	
Hypertension	195 (50.5; 45.42–55.6)
Dyslipidaemia	117 (30.3; 25.8–35.2)
Ischaemic heart disease	110 (28.5; 24.1–33.3)
Chronic heart failure	85 (22.0; 18.1–26.6)
Arrhythmia	81 (21.0; 17.1–25.5)
Cardiac valve replacement	74 (19.2; 15.4-23.5)
Rheumatic heart disease	71 (18.4; 14.7-22.7)
Cerebrovascular disease	25 (6.5; 4.3–9.5)
Drug class/drug	
Beta-blockers	236 (61.1; 56.1–66.0)
Statins	183 (47.4; 42.4–52.5)
Furosemide	182 (47.2; 42.1–52.3)
Low-dose aspirin	165 (42.7; 37.8–47.9)
Warfarin	157 (40.7; 35.8–45.8)
Angiotensin converting enzyme inhibitors	147 (38.1; 33.3–43.2)
Potassium-sparing diuretics	115 (29.8; 25.3–34.7)
Calcium-channel blockers	63 (16.3; 12.9–20.5)
Clopidogrel	60 (15.5; 12.2–19.6)
Angiotensin receptor blockers	48 (12.4; 9.4–16.2)
Nitrates	44 (11.4; 8.5–15.1)
Digoxin	31 (8.0; 5.6–11.3)
Thiazide diuretic	15 (3.9; 2.3–6.5)
Number of chronic diseases	
1–2	234 (60.6; 55.5–65.5)
≥ 3	152 (39.4; 34.5–44.5)
Number of medications	
1–4	216 (56.0; 50.8–61.0)
≥ 5	170 (44.0; 39.1–49.2)
Duration of medication use (years)	
≥ 0.25–1	75 (19.4; 15.7–23.8)
> 1–5	143 (37.0; 32.3–42.1)
> 5–10	95 (24.6; 20.5–29.3)
> 10	73 (18.9; 15.7–23.8)

Table 3 presents the distribution of responses to the MMAS-8 among the participants. Seven in 10 participants (n = 274; 71.0%; 95% CI: 66.1–75.4) reported that they never or rarely had difficulty remembering to take all their medications. Half of the respondents indicated that they felt hassled about sticking to their treatment plan (n = 194; 50.3%; 95% CI: 45.2–55.4). Over one-third of the study population reported that they had cut back or stopped their medication without telling their physicians because they felt worse (n = 140; 36.3; 95% CI: 31.5–41.3) and that they sometimes forgot to take their pills (n = 133; 34.5; 95% CI: 29.8–39.5).

**Table 3:** Distribution of responses to the eight-item Morisky medication adherence scale among the participants (n = 386)

**	*Item*	*Yes, n (%; 95% CI)*
1	Do you sometimes forget to take your pills?	133 (34.5; 29.8–39.5)
2	Over the past two weeks, were there any days when you did not take your medicine?	75 (19.4; 15.7–23.8)
3	Have you ever cut back or stopped taking your medication without telling your doctor because you felt worse when you took it?	140 (36.3; 31.5–41.3)
4	When you travel or leave home, do you sometimes forget to bring along your medications?	90 (23.3; 19.3-27.9)
5	Did you take your medicine yesterday?	375 (97.2; 94.8–98.5)
6	When you feel better, do you sometimes stop taking your medicine?	58 (15.0; 11.7–19.1)
7	Taking medication every day is a real inconvenience for some people Do you ever feel hassled about sticking to your treatment plan?	194 (50.3; 45.2-55.4)
8	How often do you have difficulty remembering to take all your medication?*	112 (29.0; 24.6–33.9)

*n (%) of once in a while, sometimes, usually, and all the time

Optimal adherence was defined as having a score of greater than six on the MMAS-8. Using this cut-off point, 49% (n = 189; 95% CI: 43.9–54.1) of respondents had optimal medication adherence and 51% (n = 197; 95% CI: 45.9–56.1) had poor medication adherence. The mean (± SD) score for the medication adherence scale was 6.1 (± 1.8). The item-total correlations were > 0.44 for each of the eight items composing the medication adherence scale. The internal consistency using Cronbach’s alpha was 0.76.

Multivariate logistic regression analysis revealed three independent variables had a significant influence on non-adherence to medication use. Respondents with high levels of education, low or middle income levels and those who taking five or more medications daily were found to be more non-adherent to medication use than those with low– intermediate education levels (p < 0.001), those with high income levels (p < 0.001), and those taking one to four medications daily (p = 0.039). Table 4 shows the results of the multivariate analysis for factors associated with high adherence to medication use.

**Table 4:** Association between non-adherence and respondents’ characteristics (n = 386)

*Characteristics*	*Poor adherence, n (%)*	*OR (95% CI)*	*p-value*
Age (years)			0.25
18–39	32 (40.5)	0.8 (0.4–1.4)	
40–49	22 (52.4)	1.1 (0.5–2.3)	
50–59	61 (61.6)	1.6 (0.9–2.8)	
≥ 60	82 (49.4)	Reference	
Educational level			< 0.001
Low–intermediate	152 (48.3)	0.3 (0.2–0.6)	
High	45 (63.4)	Reference	
Monthly income			< 0.001
Low	84 (60.0)	6.6 (3.6–12.3)	
Middle	88 (64.7)	5.7 (3.1–10.5)	
High	25 (22.7)	Reference	
Number of diseases			0.79
1–2	111 (47.4)	1.1 (0.6–1.9)	
≥ 3	86 (56.6)	Reference	
Number of medications			
1–4	99 (45.8)	0.6 (0.3–0.9)	
≥ 5	98 (57.6)	Reference	0.039

The reasons for poor medication adherence among the study participants were found to be the expensive cost of drugs (n = 210; 54.4%; 95% CI: 49.3–59.4), polypharmacy (n = 204; 52.8%; 95% CI: 47.7–57.9), lack of pharmacist’s communication with them regarding the instructions and importance of taking the drug regularly (n = 193; 50.0%; 95% CI: 44.9–55.1), lack of physician’s communication with them regarding their illness and the benefit that the medication will provide (n = 156; 40.4%; 95% CI: 35.5–45.5), bothered by side effects associated with their medications (n = 142; 36.8%; 95% CI: 32.0–41.8), and irregular availability of the drugs in their areas (n = 129; 33.4%; 95% CI: 28.8–38.4).

## Discussion

This is the first known study to be conducted among patients attending the three largest cardiac centres in Khartoum State to evaluate their level of adherence to cardiovascular medications, and to identify predictors and barriers of non-adherence. These findings would be the first step to provide a better understanding of medication adherence among cardiac patients in Khartoum State, and are valuable for policy makers and clinicians to inform future services. These results could be utilised in designing targeted strategies to improve adherence and to minimise the adverse outcomes associated with non-adherence to medications.

A very worrisome finding in this study was the highly prevalent self-reported medication non-adherence among the study population (51%), compared to that reported in two previous studies (34 and 40.4%, respectively) in Khartoum State.^15^,^16^ The current study provides more valid and meaningful results due to the use of an appropriate sample size, sampling strategy, validated MMAS-8, and its inclusion of patients with variant cardiovascular conditions in multicentre out-patient cardiac clinic settings covering the three largest cardiac referral centres in Khartoum State.

The present findings are within prevalences reported in developed and developing countries, which ranged between 31 and 60%.^4^,^5^,^9^,^14^ The high non-adherence rate demonstrated by this study is of particular concern as a potential contributing factor to poor clinical outcomes, including rehospitalisation, increased mortality rates and increased healthcare costs,^6^-^8^ and underscores the urgent need for its improvement in order for cardiac patients to derive the maximal benefit of their prescribed medications.

In our survey, levels of income and education, and polypharmacy were found to be significant predictors for non-adherence to cardiovascular medications. Medication non-adherence was significantly higher among low- and middleincome groups compared to the high-income group, which is consistent with previous studies.^10^,^14^ This finding may be attributed to the precipitous increase in living costs in Sudan during the last three years, which may have led some patients with cardiovascular diseases to consider their medication costs as a lower-priority option. Other possible reasons include the prescribing of expensive, proprietary medications instead of generics, poor health insurance coverage, and bureaucratic processes associated with insurance claims.

The current finding highlights the need for the implementation of appropriate tools to determine the patient’s ability to afford the cost of medications since many patients may be embarrassed to admit that they are having trouble affording medications; and the establishment of programmes that involve partnerships between patients, healthcare providers and payers to help patients plan for payment of medication. Also eliminating co-payments and out-of-pocket medication costs for patients with low and middle incomes may be a viable component of future interventions.

The present study shows that patients with high education levels were more non-adherent compared to those with low–intermediate education levels. This is in contrast with the findings of previous studies, which reported that poor health literacy was associated with medication non-adherence.^21^,^22^ There is considerable evidence that those with more years of education tend to have better health and healthier behaviours; however, this is not in agreement with our findings.

While the reasons for our apparently contrary findings are unclear, it may be that participants with low–intermediate education levels had better healthcare information to make appropriate health decisions and follow instructions for treatment. This better health information might be gained through health messages, which are delivered through television and/or radio. On the other hand, it is possible that respondents with high education levels are honest to admit that they are human and may not always follow instructions, while those with low–intermediate education levels may not admit that they fail to take their medications to that extent. Also an additional possible reason is that less than one-fifth of the respondents in this survey had high education levels. Hence, this finding highlights the need for further qualitative research to provide better understanding of education level as a predictor of non-adherence among cardiovascular patients in Sudan.

The current results revealed that non-adherence was significantly greatest among those taking five or more medications daily, which is consistent with previous studies.^11^,^23^ It is evident that reducing the total number of pills per day can improve medication adherence. Hence, an approach needs to be taken to reduce medication complexity through avoiding polypharmacy and using regimens with fewer daily doses. This could be achieved by maximally simplifying cardiovascular medication regimens by combining medications from three or more medication classes (e.g. aspirin, statin and antihypertensives) into a single daily ‘polypill’. The rationale is that the simpler medication regimen leads to improved adherence.^24^

These findings revealed that gender, co-morbidities and age were not statistically significant predictors for non-adherence, which contrasts with findings in some other studies, where women, elderly individuals and those who had three or more diseases had poorer adherence.^12^,^13^ A systematic review of studies conducted in developing countries revealed that patient factors such as age, gender, lifestyle and lack of access to healthcare services were not consistently associated with non-adherence.^14^ The existence of conflicting information among various studies suggests that assessment of non-adherence cannot be targeted to specific patient populations or characteristics, and the established predictors of adherence are often insufficient to identify individual patients who are likely to be non-adherent, and they should be used cautiously as a means of targeting high-risk populations.^25^

Another approach to understanding reasons for non-adherence is to identify the barriers. In contrast with predictors of adherence, barriers are restricted to potentially modifiable factors that healthcare providers and/or the healthcare system can attempt in order to reduce medication non-adherence. The top two barriers reported by the study population were the high cost of drugs and polypharmacy. These barriers reported by the patients confirm the results obtained by the Morisky eight-item scale adherence measure. These results underscore the urgent need for the development and implementation of effective strategies to overcome these barriers.

Lack of pharmacist’s communication regarding instructions and the importance of taking the medication regularly, and lack of physician’s communication regarding the disease and the benefit that the medication will provide were reported as barriers by half and two-fifths of respondents, respectively. These results highlight a call for a more active role that healthcare providers should take in assessment, education and strategic implementation efforts to promote medication adherence. It is evident that the time healthcare providers spend to achieve good patient understanding about the disease and the rationale for medication use fosters a partnership with their patients and improves medication adherence.^26^

Pharmacists need to develop counselling strategies that help patients form strong habits regarding medication use, educate patients about their medications, and provide regular follow up to ensure that patients are taking medications as directed. The value of pharmacists in adherence to cardiovascular medication was illustrated in two studies, where patients were randomised to intensive pharmacist-led intervention versus usual care. The intervention resulted in significant improvements in adherence and disease control.^27^,^28^ Sudanese pharmacists must improve their clinical knowledge and skills, demonstrate their willingness to be responsible for the patient’s drug therapy, and develop a close working relationship with other healthcare professionals.

Three in 10 responders indicated that the experienced side effects associated with their medications and irregular availability of the medication in their areas were barriers for adherence. This underscores the need for more dialogue between patients and healthcare providers about medications, including discussions about the possible side effects and management strategies, thus allowing patients to become part of the decision-making process.^29^ The irregular availability of cardiovascular medications among those resident outside Khartoum State underscores the need for multiple approaches to be used to address challenges within the healthcare system that prevent the reliable availability of essential medications, with a special focus on improving the governance of the drug-delivery system to all states of Sudan.

We acknowledge that this type of study has its limitations. It depends very much upon information given by respondents and is open to bias by inaccurate patient recall or by social desirability. The extent of truthful answers or verifying respondents’ claims is not possible in this type of study, and answers were taken at face value. A further limitation of the study is the cross-sectional nature of the data that represented one point in time and therefore does not reflect any changes in respondents’ adherence to cardiovascular medications.

## Conclusions

The findings of this study provide important information about the prevalence, predicators and barriers of medication non-adherence among out-patient cardiac patients. These results allow for important comparative work with existing and future investigations in Sudan and other developing countries. Our results showed that the use of a validated self-report instrument can provide immediate feedback to help healthcare providers identify non-adherent patients. Such brief, inexpensive tools should be more widely implemented as part of the daily care plan in the out-patient clinics.

The study findings underscore the urgent need to establish multifaceted and personalised interventions. These should incorporate affordable medications with favourable side-effect profiles, easy-to-use medication regimens with fewer daily doses, ongoing communication among patients and healthcare providers, improvement of the patient–provider partnership, and an expanding role of pharmacists through implementation of pharmaceutical care.
